# The Treatment of Cancer

**Published:** 1867-06

**Authors:** 


### *The Treatment of Cancer*

By hypodermic injections of acetic acid, as proposed by Dr. Broadbent, of London, is attracting considerable attention. The reasons given by Dr. B. for adopting this particular agent are:—

"1. This acid does not coagulate albumen, and might therefore be expected to diffuse itself throughout the tumour. The effect would not thus be limited to and concentrated in the part injected. 2. If it entered the circulation it could do no harm in any way, either by acting as a poison, or by inducing Embolism. 3. Acetic acid rapidly dissolves the walls and modifies the nuclei of cells on the microscopic slide, and might be expected to do this when the cells were *in situ*. 4. It has been applied to open cancer and to cancerous ulcerations."

On experiment, it has been found to produce little pain when thrown into the malignant structure, although acting energetically upon its components. On the contrary, it produces when injected into the healthy tissue very considerable pain but very little apparent effect upon its ultimate structure.

Dr. B. suggests rather the use of a large quantity of dilute acid than a small quantity of that which is more concentrated. It should be thrown in slowly, and any subsequent pain may be readily controlled by the ether spray. At the present time, when the hypodermic syringe and the nebulizer are in the possession of every physician, this method of treatment of a fearful affection is worthy of extensive trial.

Practically in epithelial cancer of the integument or of the tongue there is difficulty in securing retention of the fluid—a little patience however will overcome this.

The strength of the solution may be varied from equal parts of the officinal strong acid and water, to one part of the former to four or five of the latter.

The JOURNAL thinks that something more than local treatment is about always necessary in these cases. Arsenic is yet the king remedy, though another more potent is yet prayed for to dethrone it. Some profoundly acting *cell-changer* or *molecule-arranger* is demanded, or, better still, by and by we will find the cause and eradicate that.

In the common epithelial cancer or lupus, the JOURNAL advises *persistent* exclusion of air and light from the diseased part especially by the BELLADONNA—a good strong extract constantly overlaying—and this does more, it allays pain directly, lessens molecular movement and cell change, impeding the flow of blood into the part just as it does, when taken internally, in the vessels of the spinal cord. The hypodermic use of the solution of atropine might induce some of these effects but would not alone exclude the air and the light which must continuously be done in order to secure success. Even without constitutional measures, this has wrought better results than any other of the thousand and one local applications proposed—the knife and the caustic not being overlooked in this estimate.

But the arsenic is imperative. Full but gradually *diminishing* doses five days in the week and two days for rest, and thus for weeks or even months. In this manner the remedy can be used indefinitely without noxious, local, or general influence, and under the combined impression of this and the local application, the practitioner will, in an unexpectedly large proportion of cases, have the happiness of seeing the local disease gradually melt away, whilst all the time the general powers of the system will gain in vigor.

Cases which have resisted the knife or caustic may not be despaired of.

Howbeit, the vinegar remedy may have its own intrinsic sweetness worth the testing.

### *Incontinence of Urine Successfully treated by Extract of Belladonna.*

A healthy looking country girl, fourteen years old, was brought by her mother to the Metropolitan Free Hospital on the 11th of January last. She had suffered from nocturnal incontinence of urine for the last two years. Not a night passed without her wetting the bed, and to such an extent that she had been compelled to lie upon straw covered with a sheet in order to change her bed daily. She had been taken out of bed at night, scolded, and ridiculed, without any effect in making her abandon the habit. Dr. Drysdale ordered her a quarter of a grain of extract of belladona as a pill, to be taken at bedtime every night. On the 15th of January her mother came to say that she had not wet her bed since taking the medicine. Up to the 18th of January there was no return of incontinence of urine. Dr. Drysdale remarked that he had in many cases seen similar results from the use of belladona in this disease, and supposed that the drug acted by paralyzing the detrusor urinæ muscle.—*Medical News.*

### *The Ventilating Method*

In the treatment of wounds and ulcers, or Bonnisson's method, as it is sometimes termed, is worthy of more attention than it receives. The pellicle which is thus formed over the denuded surface is vastly more protective than the eminently nasty "healing ointments" and inconvenient dressings so frequently resorted to. Bathe the surface of the wound first with dilute alcohol, which of itself serves to coagulate the albuminous substance into a quasi membrane. Then with a common bellows pass currents of air upon and over it, until this is converted into a distinct thin pellicle, shining and slightly wrinkled at the periphery, and sufficiently thick and dry to bear the application of a piece of silk paper without its sticking. The ventilation should be repeated occasionally to keep up the density of the new covering, and once in a day, two or three as needed, the alcoholic lotion may be reapplied. The process is cleanly, expeditious, and more than ordinarily successful.

### *Receipts Acknowledged from:*

M. A. McLelland, $2; S. C. Maxwell, 2; A. P. Davis, 2; J. W. Craig, 5; Ph. Mathei, 2; H. V. V. Johnson, 2; B. Larimer, 2; J. C. Adkins, 2; D. S. Jenks, 2; J. R. Groesbeck, 2; W. P. Forsyth, 2; M. P. Sigworth, 2; J. L. Fuson, –; J. M. Jenkins, 2; H. R. Fowler, 2; C. Fenn, 2; H. Tombecken, 2; E. Schmidt, 2; S. C. Cravens, 2; C Luetzelschwab, 2; J Dancer, 5; P. R. Thombs, 2; C. C. Kelly, 2; E. Dooley, 2; J. S. McVan Nus, 2; H. Marshall, 2; G. Quackenbush, 2; G. W. Begg, 2; E. A. Morse, 2; C. J. Keegan, 2; F. L. Breidenstein, 2; F. McGuire, 2; W. Walker, 2; S. S. Wallihan, 2; S. P. Gilbert, 2; B. Holmes, 1; J. W. Harvey, 2; J. W. Kinman, 2; J. G. Kuener, 2; A. Hodge, 2; R. J. Brackenridge, 2; J. L. Ashbaugh, 2; D. S. Waddel, 2; C. B. Younkman, 2; J. H. Tyler, 2.

